# Nitrogen-Induced Changes in Soil Environmental Factors Are More Important Than Nitrification and Denitrification Gene Abundance in Regulating N_2_O Emissions in Subtropical Forest Soils

**DOI:** 10.3389/fpls.2022.950367

**Published:** 2022-07-12

**Authors:** Qingyan Qiu, Abubakari Said Mgelwa, Shaofei Jin, Yalin Hu

**Affiliations:** ^1^Forest Ecology and Stable Isotope Center, College of Forestry, Fujian Agriculture and Forestry University, Fuzhou, China; ^2^College of Natural Resources Management and Tourism, Mwalimu Julius K. Nyerere University of Agriculture and Technology, Musoma, Tanzania; ^3^Department of Geography, Minjiang University, Fuzhou, China

**Keywords:** nitrification, denitrification, microbial functional genes, N_2_O emissions, N application

## Abstract

Subtropical regions are currently experiencing a dramatic increase in nitrogen (N) deposition; however, the contributions of nitrification and denitrification processes to soil N_2_O emissions and the underlying mechanisms under increasing N deposition remain unclear. Therefore, a ^15^N-tracing laboratory experiment with four N application rates (0, 12.5, 25, and 50 μg ^15^N g^–1^ soil) was conducted to investigate the response of nitrification- and denitrification-derived N_2_O to N additions in an evergreen broad-leaved forest (BF) and a *Pinus* forest (PF) in the Wuyi Mountains in southeastern China. Moreover, the abundance of functional genes related to nitrification (*amoA*), denitrification (*nirK*, *nirS*, and *nosZ*), and soil properties were measured to clarify the underlying mechanisms. Results showed that nitrification-derived N_2_O emissions were generally decreased with increasing N input. However, denitrification-derived N_2_O emissions were a non-linear response to N additions, with maximum N_2_O emissions at the middle N application rate. Denitrification-derived N_2_O was the dominant pathway of N_2_O production, accounting for 64 to 100% of the total N_2_O fluxes. Soil NH_4_^+^-N content and pH were the predominant factors in regulating nitrification-derived N_2_O emissions in BF and PF, respectively. Soil pH and the *nirS* abundance contributed the most to the variations of denitrification-derived N_2_O emissions in BF and PF, respectively. Our results suggest that N application has the potential to increase the contribution of denitrification to N_2_O production in subtropical forest soils. Changes in soil chemical properties induced by N addition are more important than the abundance of nitrification and denitrification functional genes in regulating soil nitrification- and denitrification-derived N_2_O emissions.

## Introduction

Nitrogen (N) deposition is continuously increasing primarily due to increased anthropogenic fossil fuel combustion, industrialization, and N fertilizer application. Elevated N deposition is expected to not only alter N cycling in forest ecosystems but also enhance N gas loss from the soils (Lu et al., 2011; Fang et al., 2015; Niu et al., 2016; Tang et al., 2018). As one of the primary forms of N gas loss from the soils, nitrous oxide (N_2_O) is more than 298 times as effective at trapping atmospheric heat as CO_2_ and is also one of the largest stratospheric ozone-depleting substances (IPCC, 2013). Terrestrial ecosystems contribute ∼60% of the global N_2_O emissions (IPCC, 2013), of which about 15 to 55% are from tropical and subtropical forests that are believed to be the largest N_2_O terrestrial source (Solomon, 2007; Zhang et al., 2019). Moreover, N deposition in tropical and subtropical areas has been steadily increasing in recent decades (ranging from 30 to 73 kg N ha^–1^ year^–1^), and this trend is projected to continue over the coming decades (Zhang et al., 2008; Liu et al., 2013; Zhu et al., 2015).

N_2_O is produced primarily during the soil microbial processes of nitrification and denitrification, the rates of which are influenced by the inputs of N. Recent studies in subtropical forests demonstrated that the contribution of nitrification-derived N_2_O to the total N_2_O emissions was ≤50% and that of denitrification were 53 to 100% under aerobic conditions (Zhang et al., 2011; Zhu et al., 2013a; Han et al., 2018; Tang et al., 2018). In addition, the rates of denitrification have generally increased following N application (Zhu et al., 2013b; Morse et al., 2015; Niu et al., 2016; Han et al., 2018). However, few studies have focused on the response of nitrification-derived N_2_O emissions to N application, especially in subtropical forests (Han et al., 2018). Therefore, whether the response of nitrification-derived N_2_O emissions to N application is more pronounced than that of denitrification-derived N_2_O emissions or whether the contribution of denitrification to N_2_O production under elevated N input is still dominant over that of nitrification remains unclear. Thus, quantifying the underlying nitrification and denitrification contributions to the N_2_O production will help us understand the changes in the N transformation process with increasing N input in forest ecosystems.

Soil nitrification and denitrification can be influenced by different soil microbial groups, soil properties, and climate conditions (Levy-Booth et al., 2014; Cheng et al., 2019). Ammonia oxidation is not only the first but also the rate-limiting step of nitrification, which is performed by both ammonia-oxidizing archaea (AOA) and bacteria (AOB) (Chen and Peng, 2020). On the other hand, denitrification is a four-step microbial facilitated reduction process, whereby NO_3_^–^ is reduced to NO_2_^–^, NO, N_2_O, and eventually to N_2_. The first step for the gas production during denitrification is the reduction of NO_2_^–^ to NO, which is catalyzed by nitrite reductases encoded by *nirK* and *nirS* genes (Levy-Booth et al., 2014; Hu et al., 2015). N_2_O can be further reduced to N_2_ by nitrous oxide reductases, which are encoded by *nos*Z genes (Levy-Booth et al., 2014). While the importance of nitrification and denitrification microorganisms in regulating soil N_2_O emission has been widely accepted (Chen and Peng, 2020), to what extent the abundance of nitrifier and denitrifier functional gene can be a good predictor of N_2_O emissions under elevated N inputs remain unclear. Moreover, several recent studies have reported that soil environmental factors (e.g., NH_4_^+^, NO_3_^–^, pH, TN/TP, and TC/TN) can explain better the changes in soil nitrification and denitrification activities than soil nitrification and denitrification functional genes in fertilized forest soils (Pett-Ridge et al., 2013; Shcherbak et al., 2014; Tang et al., 2018, 2019). Although tropical and subtropical forest soils are the largest N_2_O terrestrial sources (Solomon, 2007; Soper et al., 2018; Zhang et al., 2019), only a few studies so far in these regions have attempted to directly link the nitrification- and denitrification-derived N_2_O emissions in fertilized soils to N functional genes (Tang et al., 2018, 2019), and more importantly, to investigate the relative contribution of soil biotic and abiotic factors on the nitrification- and denitrification-derived N_2_O emissions. Greater insight into the regulation of these processes can help us understand the underlying mechanisms and adopt management practices to lower N_2_O emissions.

To evaluate the changes in soil N_2_O emissions resulting from nitrification and denitrification processes under N application in subtropical forests and clarify the underlying mechanisms, we carried out a ^15^N-NO_3_^–^ and ^15^N-NH_4_^+^ labeling experiment under laboratory conditions. Mineral soils were collected from two subtropical forests, an evergreen broad-leaved forest (BF), and a *Pinus* forest (PF), which are located in the Wuyi Mountains in southeastern China. The evergreen BF was chosen because it is a product of the monsoon climate and it represents the climax vegetation type of subtropical regions (Zhou et al., 2013). *Pinus* forest was selected because of its strong contribution to afforestation purposes since the 1980s. It is worth highlighting that the selected pine species have a strong ability to adapt to poor soil conditions in the hilly red soil region of south China, which faced moderate to intense soil and water loss challenges in the 1980s. The forests have been widely planted since that time and account for about 30.5% of the subtropical forests in south China (Cao et al., 2015). Many previous studies have demonstrated that broadleaf/deciduous species produce more N_2_O than conifers due to a large difference in the production and reduction rates of N_2_O under the broadleaf/deciduous species (Zhang et al., 2008; Qin et al., 2019). However, some studies reported that coniferous stands emit more N_2_O than the adjacent deciduous stands that were affected by N deposition. Therefore, the role of different forest types (or tree species) in regulating N_2_O emissions remains unclear.

This study aimed to (1) investigate the responses of nitrification- and denitrification-derived N_2_O emissions to elevated N inputs, (2) clarify the dominant factors that control nitrification- and denitrification-derived N_2_O emissions, and (3) compare the differences in nitrification- and denitrification-derived N_2_O emissions between the BF and PF. We hypothesized that (1) nitrification- and denitrification-derived N_2_O emissions would be enhanced with the increased N application and that denitrification may contribute more to the increase in N_2_O emissions than nitrification, (2) the abundances of functional genes could be more important than soil environmental factors in regulating nitrification- and denitrification-derived N_2_O emissions, and (3) the nitrification- and denitrification-derived N_2_O emissions would be greater in the BF than the PF.

## Materials and Methods

### Study Site Descriptions and Soil Sampling Procedures

The experimental soil samples were collected at the Wuyishan National Nature Reserve (WNNR), located in Fujian Province (27°33′–27°54′N, 117°27′–117°51′E) in southern China. The climate of the WNNR (area: 56,527 ha) is humid monsoon, which is mainly characterized by the mean annual precipitation and temperature of 2,000 mm and 15°C, respectively, as well as 83.5% relative humidity and 100 fog days year^–1^ (Xu et al., 2010; Wang et al., 2013). The WNNR has two typical vegetation types, that is, evergreen BF and pine forest (PF). The BF and PF are located at about 550 m a.s.l. and 1,100 m a.s.l., respectively. While *Castanopsis carlesii* Hay and *Castanopsis eyrie* Tutch are the dominant species in the BF, *Pinus taiwanensis* Hayata is the dominant species in the PF (Xu et al., 2010; Lyu et al., 2019). Background atmospheric N deposition in the WNNR is about 8 kg N ha^–1^ year^–1^ (Xu et al., 2016; Gurmesa et al., 2022).

In April 2018, three 20 m × 20 m plots without N addition (plots were about 10 m away from each other) were randomly established in each vegetation type, and soil samples were collected with a soil core sampler (8 cm in diameter) from the surface soil layer (0–20 cm) after litter removal. Ten soil cores were randomly collected from each plot and there were a total of 30 soil cores collected in each forest stand. Soil samples from the same forest type were thoroughly mixed to form a composite sample. All soil samples were sieved through a 2-mm mesh sieve and were then divided into two sub-samples. The first sub-sample was maintained at 4°C for 1 week before being used for the incubation experiment. The second sub-sample was air-dried for the measurements of soil chemical properties. The soil at both sites is classified as yellow-red soil based on the Chinese Soil Classification System, equivalent to an Ultisol according to the United States Department of Agriculture Soil Taxonomy classification system (Lyu et al., 2019). Soil chemical properties were shown in Table 1. The concentrations of soil SOC, TN, NH_4_^+^-N, and NO_3_^–^-N in the PF were significantly higher than those in the BF, whereas soil pH and δ^15^N were lower in the PF and no significant difference was found in the soil C/N ratio in these two forest soils (Table 1).

**TABLE 1:** Soil chemical properties in the BF and PF.

Forest type	SOC (%)	TN (%)	C/N	δ^15^N (‰)	pH	NH_4_^+^-N (mg kg^–1^)	NO_3_^–^-N (mg kg^–1^)
BF	4.10 ± 0.07b	0.21 ± 0.01b	19.80 ± 0.47a	10.84 ± 1.24a	4.80 ± 0.03a	91.89 ± 1.13b	9.40 ± 0.06b
PF	5.67 ± 0.20a	0.27 ± 0.01a	20.63 ± 0.12a	9.97 ± 1.56b	4.72 ± 0.01b	126.66 ± 3.07a	15.37 ± 0.56a

*BF and PF indicate the broad-leaved forest and Pinus forest, respectively. Values are means ± standard error, n = 3. Different lowercase letters in the same column represent the difference was significant at P < 0.05.*

### ^15^N-Tracing Experiment and Gas Sampling Procedures

This study used a completely randomized factorial design experiment that consisted of four N application rate treatments (0, 12.5, 25, and 50 μg ^15^N g^–1^ soil equivalent to 0, 16, 32, and 64 kg N ha^–1^ year^–1^). We applied N as ^15^NH_4_NO_3_ (99.10 atom %) and NH_4_^15^NO_3_ (99.21 atom %). The rates of N application were in the range of those of N deposition (10 to 73 kg N ha^–1^ year^–1^) in subtropical forests (Zhang et al., 2011, 2019). For each forest soil type, 120 glass jars (500 mL) were prepared and divided into two groups: one group comprised of 60 glass jars containing ^15^NH_4_NO_3_ (4 N addition rates × 3 replications per N application rate × 5 destructive soil sampling) and the other group consisted of 60 glass jars containing NH_4_^15^NO_3_. A total of 30 g (oven-dry basis) of fresh soil was weighed into each glass jar and pre-incubated for 7 days at 25°C to allow the recovery of soil microbial activities. Then, ^15^NH_4_NO_3_ or NH_4_^15^NO_3_ was dissolved in 4 mL deionized water and added to the soil at a rate of 0, 12.5, 25, and 50 μg ^15^NH_4_^+^-N g^–1^ soil or ^15^NO_3_^–^-N g^–1^ soil. Soil without N addition was regarded as a control and was added with the same volume of deionized water to ensure its similarity in soil moisture content with the N-added soil. The soils were then incubated for 15 days at 25°C and their moisture content was maintained at 60% water-holding capacity throughout the entire experiment. Soils were corrected for water loss by weighing each glass jar plus soil every week and deionized water was added as required to maintain constant soil moisture. During gas sampling, three glass jars at each level of N treatment were randomly selected. The glass jars were closed with the gas-tight rubber lids, and gas sampling tubes were fixed into the middle of the rubber lids to enable the collection of the gas samples. Gas samples (20 mL) were collected from each glass jar with a 3-way stopcocks syringe on days 1, 2, 4, 9, and 15 after N application. Five millimeters were taken from each gas sample to determine the N_2_O concentration (Shimadzu, GC-2014C, Japan) and the remaining 15 mL were used to determine the N_2_O isotopic composition (IRMS, Isoprime 100, United Kingdom). Before each gas sampling, the glass jars were opened and flushed with ambient air for approximately 30 min and then resealed with stoppers for 30 min.

### Quantification of the Contributions of Nitrification and Denitrification to N_2_O Emissions

The fractions of N_2_O derived from denitrification (*FD*) and nitrification (*FN*) processes were calculated using the following equations from Stevens et al. (1997):


(1) FD=(a-ma)n/(a-da)n

(2) F⁢N=1-F⁢D

where *a*_m_ is the ^15^N atom % of N_2_O, *a*_n_ is the ^15^N atom % of N_2_O from the nitrification process (assumed to be equivalent to ^15^N atom % of the soil NH_4_^+^), and *a*_d_ is the ^15^N atom % of N_2_O from denitrification (assumed to be equivalent to ^15^N atom % of the soil NO_3_^–^).


The⁢atom⁢percent⁢of⁢N15⁢is⁢defined⁢as⁢N15⁢atom%

(3) =N15/(N15+N14)×100%

(4) Denitrification-derived⁢N⁢O2⁢flux=F⁢D×N⁢O2⁢flux

(5) Nitrification-derived⁢N⁢O2⁢flux=F⁢N×N⁢O2⁢flux

### Soil Inorganic N, Net Nitrification Rate, Dissolved Organic Carbon, Dissolved Organic Nitrogen, and pH Measurements

Soil samples were also collected after gas sampling. Five times destructive soil sampling was used for the analysis of the soil NH_4_^+^ and NO_3_^–^ contents, as well as the δ^15^N values of soil NH_4_^+^ and NO_3_^–^. Dissolved organic carbon (DOC) and nitrogen (DON) contents and soil pH were also determined at the end of the experiment. Soil NH_4_^+^ and NO_3_^–^ contents were extracted using 2 mol L^–1^ KCl for 1 h and then the extracted solutions were divided into two parts. One part was reacted with sodium salicylate (for NH_4_^+^ solution) and hydrazine sulfate solution (for NO_3_^–^ solution) and measured, respectively, at 660 and 550 nm using a discrete wet chemistry analyzer (SmartChem 200, AMS Alliance, Italy) to determine the concentrations of soil NH_4_^+^-N and NO_3_^–^-N. The other part was used for ^15^N measurements by distillation using MgO and Devarda’s alloy (Zhang et al., 2011). The DOC in the soil was extracted with 2 mol L^–1^ KCl at 1:5 w/v and measured using a TOC-TN analyzer (Shimadzu TOC, Japan). The soil DON concentration was determined by calculating the difference between soil total dissolved nitrogen (2 mol L^–1^ KCl extracted at 1:5 w/v) and soil mineral nitrogen (Jones and Willett, 2006). Soil pH was measured at a 1:2.5 soil to solution ratio using a pH detector (PHS-3C, Sheng Ci, Shanghai, China).

### Quantification of the *amoA*, *nirK*, *nirS*, and *nosZ* Gene Abundances

At the end of the incubation experiment, soil samples for molecular analyses were immediately stored at −80°C before the aforementioned analysis. DNA was extracted from 0.3 g of frozen soils using a Mo Bio PowerSoil DNA Isolation Kit (Mo Bio Laboratories, Carlsbad, CA, United States) according to the manufacturer’s protocol. The concentration and purity of the extracted DNA were determined with a spectrophotometer at 260 nm (NanoDrop Technologies, United States). All extracted soil DNA samples were stored at −80°C before analysis. Quantitative polymerase chain reaction (qPCR) was used to estimate the abundance of nitrification and denitrification functional genes (*amo*A, *nirK*, *nirS*, and *nosZ*) using a real-time PCR detection system. The PCR primers used in this study are listed in Supplementary Table 1. The 10-fold serially diluted plasmids carrying each target gene were subjected to real-time PCR assays in triplicate to generate a standard curve. The qPCR efficiencies for AOA-*amoA*, AOB-*amoA*, *nirK*, *nirS*, and *nosZ* were 0.85, 0.94, 0.96, 0.91, and 0.87, respectively. The corresponding determination coefficients of the standard curve for AOA-*amoA*, AOB-*amoA*, *nirK*, *nirS*, and *nosZ* were 0.998, 0.993, 0.990, 0.999, and 0.996, respectively.

### Statistical Analyses

The gene copy numbers were log-transformed to meet the homogeneity of variance requirements. Three-way ANOVA was used to examine the effects of forest type, N application rate, sampling date, and their interactions on soil N_2_O flux, nitrification-, and denitrification-derived N_2_O emissions, as well as on soil NH_4_^+^-N, NO_3_^–^-N and their ratio (NH_4_^+^-N/NO_3_^–^-N). Two-way ANOVA with Duncan’s test was performed to determine the effects of forest type, N application rate, and their interactions on the soil properties (soil pH, DOC, and DON) and the abundances of soil nitrification and denitrification functional genes (*amoA*, *nirK*, *nirS*, and *nosZ*). All of the above statistical analyses were performed using SPSS 16.0 (SPSS Inc., Champaign, IL, United States).

To estimate the direct and indirect effects of soil biotic and abiotic factors on soil nitrification- and denitrification-derived N_2_O emissions in the BF and MF, structural equation models (SEM) were performed based on our knowledge of soil biotic and abiotic properties in relation to soil nitrification- and denitrification-derived N_2_O. Before the SEM analyses, the units of the predictor and dependent parameters were adjusted to obtain comparable parameter variances according to the standardized method. The quality of the SEM model was assessed by using a low χ^2^ value and *P* > 0.05, the root-mean-square error of approximation (RMSEA, the RMSEA ≤0.08 indicates a model fit), and the model fit index (GFI > 0.90). All SEM analyses were using Amos 17.0 (IBM, SPSS, NY, United States).

## Results

### Effects of N Addition on Total and Nitrification- and Denitrification-Derived N_2_O Emissions

**FIGURE 1:**
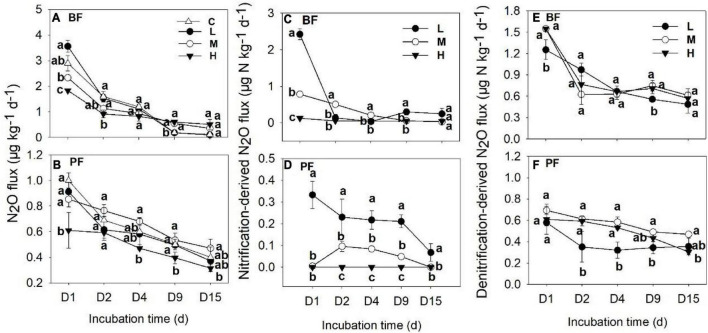
Dynamics of soil N_2_O fluxes **(A,B)**, nitrification- **(C,D)**, and denitrification-derived **(E,F)** soil N_2_O fluxes under different treatments. Values are means ± standard error (*n* = 3). Different lowercase letters for the same incubation time represent significant differences between different treatments. C, L, M, and H represent N application rates of 0, 12.5, 25, and 50 μg ^15^N g^–1^ soil, respectively. BF and PF indicate broad-leaved forest and *Pinus* forest, respectively.

**FIGURE 2:**
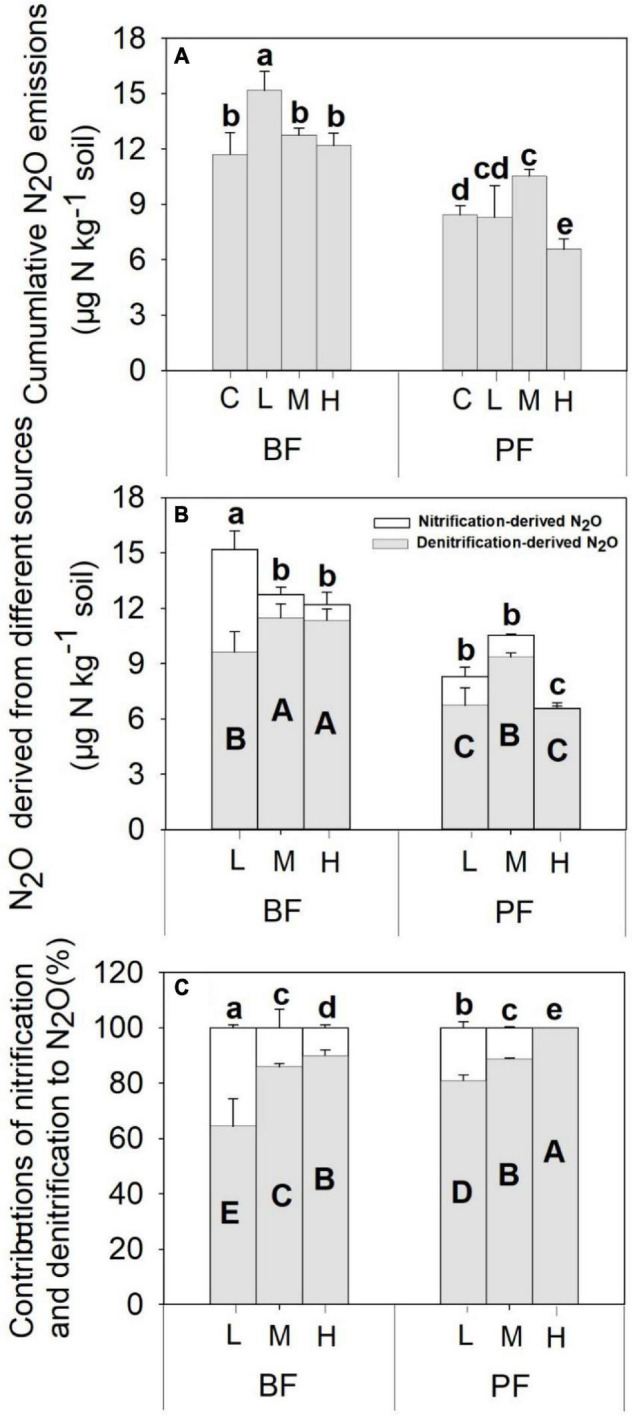
Nitrification-**(A)** and denitrification-**(B)** derived soil N_2_O emissions, as well as the contributions of nitrification and denitrification to total soil N_2_O emissions **(C)**, under different treatments. Values are means ± standard error (*n* = 3). Different lowercase letters indicate that the nitrification-derived N_2_O differed significantly between treatments (*P* < 0.05). Different uppercase letters indicate that the denitrification-derived N_2_O differed significantly between treatments (*P* < 0.05). C, L, M, and H represent N application rates of 0, 12.5, 25, and 50 μg ^15^N g^–1^ soil, respectively. BF and PF represent the broad-leaved forest and *Pinus* forest, respectively.

Soil N_2_O fluxes decreased with increasing incubation period (Figure 1) and were significantly affected by forest type, N application rate, sampling date, and their interactions (Table 2). The N_2_O flux values in the BF ranged from 0.05 to 3.85 μg N kg^–1^ day^–1^ throughout the entire incubation period (Figure 1), with the average fluxes across all the treatments being about 2.4 times greater than those in the PF (Figure 1). Non-linear responses of the soil N_2_O fluxes to N addition were observed in both the BF and the PF. Specifically, the highest amount of soil N_2_O emissions was noted at low and moderate N applications in the BF and PF, respectively (Figure 2A).

**TABLE 2:** *P*-values from Repeated-measure ANOVA of the effects of forest type (*FT*), N application rates (*N*), sampling date (*D*), and their interaction on the N_2_O fluxes, nitrification-, and denitrification-derived N_2_O emissions, soil NO_3_-N, and NH_4_^+^-N, *n* = 3.

	*FT*	*N*	*D*	*FT* * *N*	*FT* * *D*	*N* * *D*	*FT* * *N* * *D*
N_2_O fluxes	<0.001	<0.01	<0.001	0.157	<0.001	<0.001	<0.001
Nitrification-derived N_2_O	<0.001	<0.001	<0.001	<0.001	<0.001	<0.001	<0.001
Denitrification-derived N_2_O	<0.001	<0.05	<0.001	0.123	<0.001	0.274	<0.05
NO_3_-N	<0.001	<0.001	<0.001	0.605	<0.001	<0.001	<0.001
NH_4_^+^-N	<0.001	<0.001	<0.001	0.554	<0.001	<0.001	<0.01
NH_4_^+^-N/NO_3_^–^-N	0.222	<0.001	<0.001	<0.001	<0.001	<0.001	<0.01

The application rates of N fertilizer altered not only the total amount of soil N_2_O fluxes but also the nitrification- and denitrification-derived N_2_O fluxes (Figures 1, 2). The nitrification- and denitrification-derived N_2_O emissions were significantly higher in the BF than in the PF (Figure 2B). The nitrification-derived N_2_O fluxes were in the range of 0.03 to 2.66 μg N kg^–1^ day^–1^ and 0 to 0.45 μg N kg^–1^ day^–1^ in the BF and PF, respectively (Figures 1C,D), and these fluxes decreased significantly as N application rates increased in both forests. At high rates of N addition, the decrease in nitrification-derived N_2_O fluxes was approximately 84.6% in the BF and 100% in the PF relative to those observed at low N addition rates (Figure 2B). The denitrification-derived N_2_O fluxes were in the range of 0.4 to 1.7 μg N kg^–1^ day^–1^ and 0.2 to 0.8 μg N kg^–1^ day^–1^ in the BF and PF, respectively (Figures 1E,F) and maximum average of denitrification-derived N_2_O emissions was observed in middle N application rate in both BF and PF (Figure 2B). Moreover, denitrification was the dominant pathway of soil N_2_O production in the present study, accounting for 64 to 90% and 81 to 100% of the total N_2_O fluxes in the BF and PF, respectively (Figure 2C).

### Effects of N Addition on Soil Inorganic N, pH, Dissolved Organic Carbon, and Dissolved Organic Nitrogen

**FIGURE 3:**
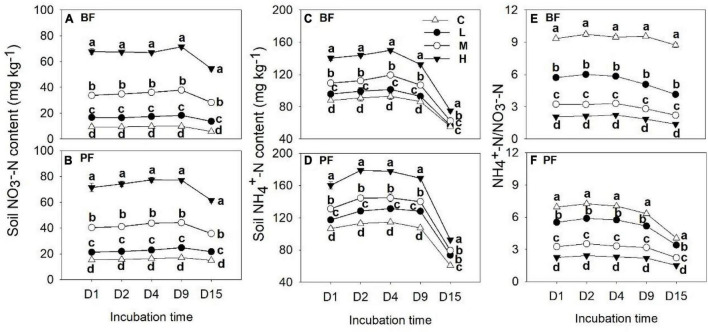
Dynamics of soil NO_3_^–^-N **(A,B)** and NH_4_^+^-N contents **(C,D)** and soil NH_4_^+^-N to NO_3_^–^-N ratio **(E,F)** under different treatments. Values are means ± standard error (*n* = 3). Different lowercase letters indicate that the soil NO_3_^–^-N and NH_4_^+^-N contents and their ratios differed significantly among treatments at each sampling time (*P* < 0.05). C, L, M, and H represent N application rates of 0, 12.5, 25, and 50 μg ^15^N g^–1^ soil, respectively. BF and PF represent the broad-leaved forest and *Pinus* forest, respectively.

**FIGURE 4:**
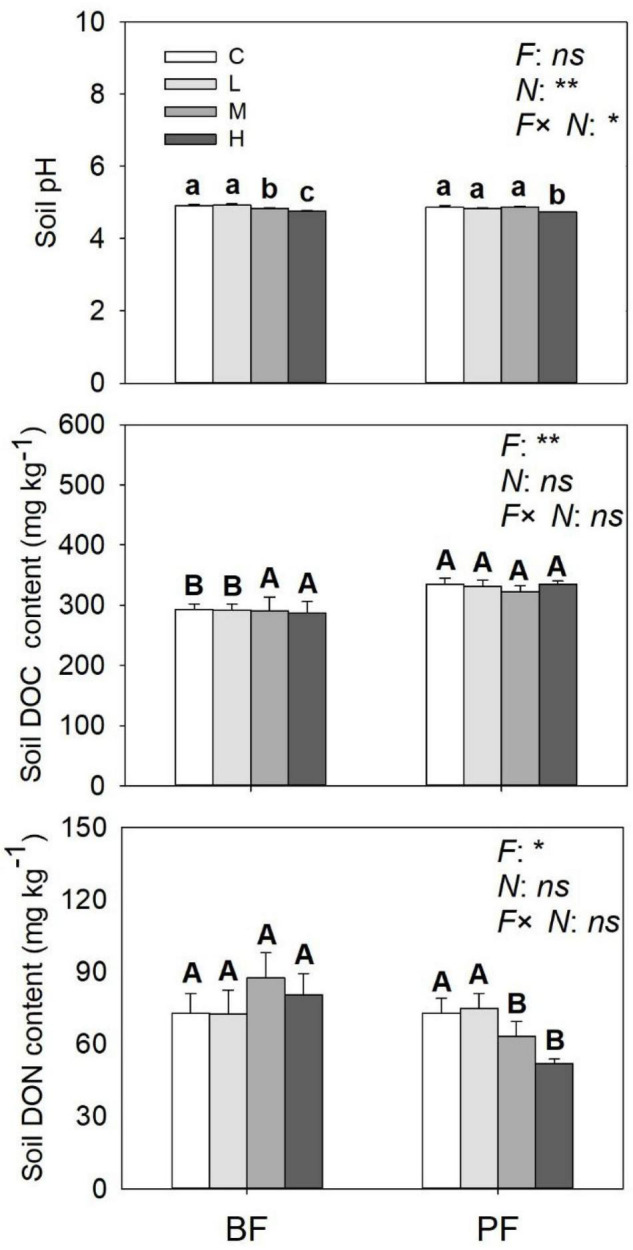
Soil pH, soil DOC, and DON contents under different treatments. Values are means ± standard error (*n* = 3). Different lowercase letters indicate that soil pH, soil DOC, and DON contents differed significantly between treatments (*P* < 0.05). Different uppercase letters indicate that soil pH, soil DOC, and DON were significantly different between the two forest soils (*P* < 0.05). C, L, M, and H represent N application rates of 0, 12.5, 25, and 50 μg ^15^N g^–1^ soil, respectively. BF and PF represent the broad-leaved forest and *Pinus* forest, respectively. *F*, *N*, and *F* × *N* represent the effects of forest type, N application, and their interactions on these indicators. * and ** represent the effects of forest type or N application or their interactions on these indicators were significant at *P* < 0.05 and *P* < 0.01, respectively. ns indicate their effects were not significant at *P* < 0.05.

Soil NO_3_^–^-N and NH_4_^+^-N contents were significantly affected by forest type and N application rate (Table 2). The soil NO_3_^–^-N and NH_4_^+^-N contents increased from day 1 to day 9 and then decreased significantly on the last day of the incubation experiment (Figure 3). These soil inorganic N contents increased with increasing N application rates, and they were significantly higher in the PF than in the BF (Figures 3A–D). Soil NH_4_^+^-N contents in the control treatments were, respectively, ∼9- and ∼7-fold greater in the BF and PF compared with soil NO_3_^–^-N contents (Figures 3E,F). Moreover, the ratio of soil NH_4_^+^-N to soil NO_3_^–^-N decreased with increasing N inputs. High N application significantly decreased soil pH in both forests. While high N addition significantly decreased soil DON content in PF, no significant effect of N addition on soil DOC was observed in this forest type (Figure 4).

### Effects of N Addition on the Abundances of Nitrification and Denitrification Functional Genes

**FIGURE 5:**
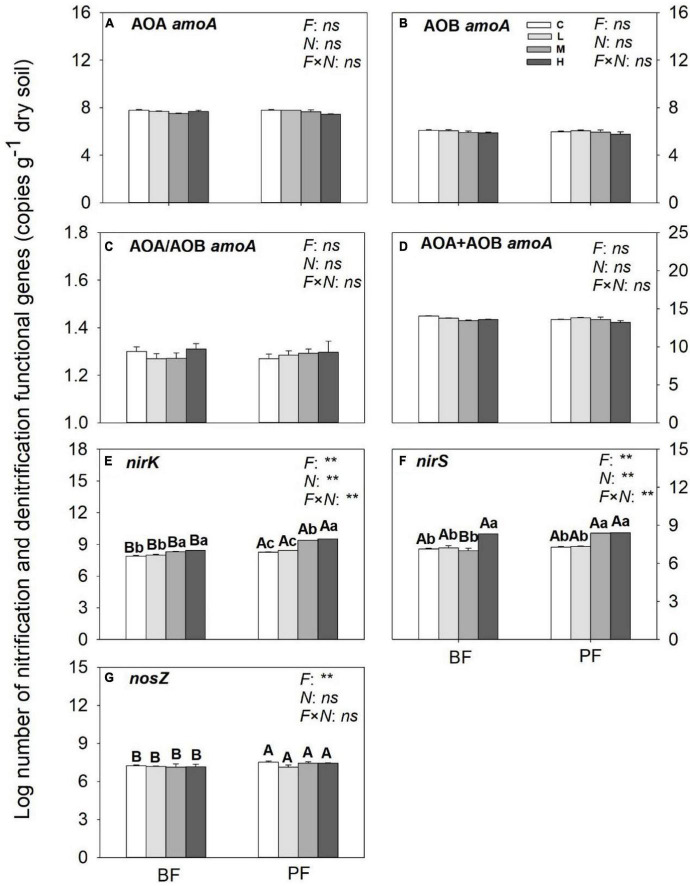
Abundances of nitrification **(A–D)** and denitrification **(E,F)** functional genes under different treatments. Values are means ± standard error (*n* = 3). Different lowercase letters indicate that the functional gene abundance differed significantly among treatments (*P* < 0.05). C, L, M, and H represent N application rates of 0, 12.5, 25, and 50 μg ^15^N g^–1^ soil, respectively. BF and PF represent the broad-leaved forest and *Pinus* forest, respectively. *F*, *N*, and *F* × *N* represent the effects of forest type, N application, and their interactions on these indicators. ^**^ represent the effects of forest type or N application or their interactions on these indicators were significant at *P* < 0.05 and *P* < 0.01, respectively. ns indicate their effects were not significant at *P* < 0.05.

While the abundances of AOA- and AOB-*amoA* genes in the BF and PF were not significantly affected by N inputs (Figures 5A–D), those of *nirK* and *nirS* genes were increased substantially by N inputs (Figures 5E,F). The increase in abundance of *nirK* genes in the PF (12%) was greater than that in the BF (5%), whereas the increase in abundance of *nirS* genes in the BF (15%) was slightly higher compared with that in the PF (14%). Inputs of N had no significant effect on *nosZ* gene abundance (Figure 5G).

### Controlling Factors of Nitrification- and Denitrification-Derived N_2_O Emissions

**FIGURE 6:**
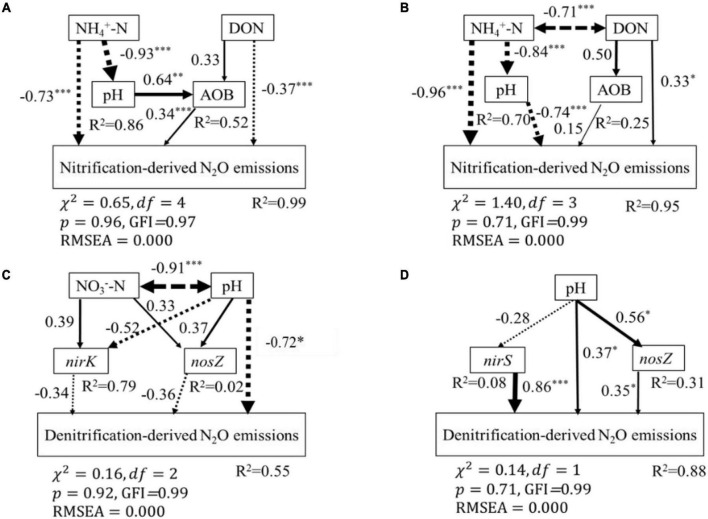
Structural equation modeling (SEM) analysis of the effects of soil biotic and abiotic properties on soil nitrification-derived N_2_O emissions in BF **(A)**, PF **(B)**, and on soil denitrification-derived N_2_O emissions in BF **(C)** and PF **(D)**, respectively. Continued and dashed arrows indicated positive and negative relationships in a fitted SEM, respectively. Arrow line thickness indicates the strength of the causal relationship. Numbers adjacent to arrows represented standardized path coefficients of the relationships (*P < 0.05, ^**^P < 0.01, ^***^P < 0.01). Double-headed arrows represented covariance between variables. The total variation explained by the model is indicated by *R*^2^.

According to the SEM, soil properties (soil NH_4_^+^-N, NO_3_^–^-N, DON, and pH) and nitrification and denitrification functional genes (AOA-*amoA*, AOB-*amoA*, *nirK*, *nirS*, and *nosZ*) explained 99 and 95% of the variance observed in nitrification-derived N_2_O emissions in the BF and PF, respectively (Figures 6A,B). These variables also could explain 55 and 88% of the variance of denitrification-derived N_2_O emissions in the BF and PF, respectively (Figures 6C,D). The dominant factor in regulating soil nitrification-derived N_2_O emission in the BF and PF was soil NH_4_^+^-N (standardized total coefficient = –0.93, *P* < 0.001) and soil pH (standardized total coefficient = −0.74, *P* < 0.001), respectively, (Table 3). The dominant factors in regulating soil denitrification-derived N_2_O in BF and PF were significantly differently (Figures 6C,D and Table 3). In the BF, it was soil pH. However, in the PF, the dominant regulatory factor was *nirS* (Table 3).

**TABLE 3:** Standardized total effects of each explanatory variable on nitrification- and denitrification-derived N_2_O emissions.

	Nitrification-derived N_2_O emissions		Denitrification-derived N_2_O emissions	
	Explanatory variable	Standardized total effects	Explanatory variable	Standardized total effects
BF	NH_4_^+^-N	–0.93	pH	–0.85
	AOB	0.34	*nosZ*	–0.36
	DON	–0.26	*nirK*	–0.34
	pH	0.22	NO_3_^–^-N	–0.25
PF	pH	–0.74	*nirS*	0.86
	NH_4_^+^-N	–0.54	*nosZ*	0.35
	DON	0.41	pH	0.33
	AOB	0.15		

## Discussion

### Effects of N Addition on Nitrification- and Denitrification-Derived N_2_O Emissions

In the present study, soil N_2_O fluxes responded non-linearly to N addition. Low- and middle-level N application rates increased soil N_2_O emissions which might be due to N application and increased N availability (Figure 3) for nitrifying and denitrifying microorganisms. However, high N input had no significant effect on soil N_2_O emission from BF or even decreased soil N_2_O emission from PF (Figure 2B). Similarly, previous studies carried out in a subtropical montane forest also reported that short-term low and medium N addition favored N_2_O production but this effect became weaker or even decreased N_2_O emissions at high N addition (Han et al., 2018). A possible reason may be that the positive effect of high N application on denitrification-derived N_2_O in both forests was partially offset by its negative effect on nitrification-derived N_2_O (Figure 2B). Another explanation may be that the consumption of N_2_O may be greater than the production of N_2_O at high N treatment (Senbayram et al., 2012).

The application of N not only affects total soil N_2_O emissions but also the contribution of nitrification and denitrification processes to soil N_2_O production. In the present study, the average nitrification-derived N_2_O emissions under different N treatments ranged from 0 to 0.37 μg N kg^–1^ day^–1^ (Figures 1C,D), which is similar to a previous study in subtropical forests [0.06–0.40 μg N kg^–1^ day^–1^, Zhang et al. (2011)]. Over 15 days of incubation, nitrification-derived N_2_O contributed 0 to 36% of the total N_2_O emissions (Figure 2). This range is within the range (<50%) that has been documented in previous studies carried out in the subtropical forest with similar N application rates (Zhang et al., 2011; Han et al., 2018). In contrast to our first hypothesis, our result showed a decrease in the contribution of the nitrification process to the total N_2_O emissions following N application (Figure 3), and the magnitude of decrease was about 80 to 100% across the two forests. Deppe et al. (2017) also found that N addition can significantly decrease the contribution of nitrification to N_2_O production in a Haplic Luvisol soil, but the magnitude of decrease (7 to 21%) was not as pronounced as the one observed in the present study. One possible explanation for this phenomenon may be that N application decreased soil pH (Figure 4A), and soil nitrification rates have been demonstrated to be relatively low in acidic soils and decrease with decreasing soil pH (Cheng et al., 2013; Wang et al., 2020).

In contrast to the response of nitrification-derived N_2_O emissions to N input as explained above, the highest denitrification-derived N_2_O emissions occurred at the middle N application rate in both BF and PF, and the denitrification process is the dominant pathway for the production of soil N_2_O emission, accounting for 64 to 100% of the total N_2_O fluxes (Figure 3C). Previous studies carried out across subtropical forests reported that 53 to 100% of the total N_2_O emissions were derived from denitrification (Zhang et al., 2011; Zhu et al., 2013a; Han et al., 2018). Moreover, the relative contributions of denitrification to N_2_O emissions in forest soils were found to increase significantly with increasing N application (Zhu et al., 2013a; Morse et al., 2015; Niu et al., 2016), with the magnitude of increase ranging from 51 to 130% in a subtropical forest (Zhu et al., 2013a). The significantly higher relative contribution of denitrification to total N_2_O production in the PF compared with the BF in the present study can be partly attributed to the greater decrease in nitrification-derived N_2_O emissions in the PF (100%) than in the BF (80%).

Moreover, both nitrification- and denitrification-derived N_2_O emissions were significantly higher in the BF relative to the PF, which indicated that the BF was a more important N_2_O emission source than the PF. This is consistent with our third hypothesis. A study by Zhang et al. (2008) in southern China also reported that the emissions of N_2_O from a mature monsoon evergreen BF were significantly higher compared to those from a PF. The authors attributed their observation to the higher soil N availability in the monsoon evergreen BF. However, their explanation cannot be extended to our findings because the BF had significantly lower soil available N content than the PF (Figure 3). Considerable differences in the amount of N_2_O emissions among the investigated forest soils might be attributed to the differences in tree species, or more specifically, their influences on the soil’s abiotic and/or biotic properties (Butterbach-Bahl et al., 1997; Qin et al., 2019). It has been reported that trees in BF are associated with arbuscular mycorrhizal fungi, which can promote soil microbial communities with higher N cycling potential and activity relative to microbial communities in soils dominated by the trees associated with ectomycorrhizal fungi in the PF (Mushinski et al., 2021).

Overall, our results supported part of our first hypothesis as the denitrification process was the dominant pathway of soil N_2_O production. However, the denitrification-derived N_2_O emissions were observed as non-linear responses to N application in both forests. In addition, the nitrification-derived N_2_O emissions decreased with increasing N application in both BF and PF, which is in contrast with our first hypothesis. An explanation for this phenomenon is presented in section “Controlling Factors Related to Nitrification-Derived N_2_O Emissions in Forest Soils.”

### Controlling Factors Related to Nitrification-Derived N_2_O Emissions in Forest Soils

Soil NH_4_^+^-N content and soil pH contributed the most to the variations of nitrification-derived N_2_O emissions in the BF and PF, respectively (Figure 6 and Table 3). Their effect was more pronounced than the effects of AOB-*amoA* on nitrification-derived N_2_O emission. Therefore, in contrast to our second hypothesis, our results suggest that N-induced changes in soil environmental factors were more important than the abundance of *amo*A genes in regulating nitrification-derived N_2_O emissions. Similarly, Tang et al. (2019) found that soil environmental factors (especially soil NH_4_^+^ content) accounted for 50 to 77% of the variation in potential nitrification activities, which was better explained than nitrification functional genes in fertilized soils. In the BF, high soil NH_4_^+^-N concentrations not only had a direct negative effect on nitrification-derived N_2_O emission but also had an indirect negative effect *via* influencing soil pH and the abundance of AOB (Figure 6A). The reasons for high soil NH_4_^+^-N contents reducing nitrification-derived N_2_O emission may be as follows: ammonia oxidizers are unable to tolerate high NH_4_^+^-N contents (>100 mg N kg^–1^ soil) in acidic soils (Gubry-Rangin et al., 2010; Carey et al., 2016; Farquharson, 2016; Breuillin-Sessoms et al., 2017; Li et al., 2018; Liu et al., 2018). In the current study, the average soil NH_4_^+^-N content in N-amended soils was much higher than not only the aforementioned 100 mg N kg^–1^ soil but also other soil NH_4_^+^-N content averages that have been reported in previous studies in various subtropical fertilized forest soils (ranging from 1 to 50 mg N kg^–1^ soil) (Han et al., 2018; Tang et al., 2018, 2019; Wang et al., 2018). This indicates the lack of efficient oxidation of NH_4_^+^ to NO_3_^–^ during nitrification that has led to lower nitrification-derived N_2_O emissions in N-amended soils (Farquharson, 2016; Wang et al., 2020).

Soil NH_4_^+^-N content also had a direct significant effect on nitrification-derived N_2_O emission in the BF, but its total effect was offset by its indirect effect on soil pH (Figure 6B). This resulted in soil pH playing a more important role in regulating nitrification-derived N_2_O emissions in the PF (Table 3). Previous studies have reported that soil with a lower pH support less microbial diversity and lower N-cycle potential (Cheng et al., 2019; Mushinski et al., 2021). A meta-analysis reports that N addition dramatically decreases the ratios of fungi to bacteria and microbial C to N on an average by 10 and 8%, respectively (Zhou et al., 2017). In addition, the nitrification rate is generally decreased with decreasing soil pH, and its rate can be inhibited in soil with a pH lower than 5 (Zhao et al., 2018; Cheng et al., 2019). This could explain why the nitrification-derived N_2_O emission at high N addition in the PF is close to zero (Figure 2B). Overall, in terms of nitrification-derived N_2_O emissions, changes in soil environmental factors (e.g., soil NH_4_^+^-N and pH) induced by N application play more important roles than changes in the abundance of AOA and AOB in controlling its emission.

### Controlling Factors Related to Denitrification-Derived N_2_O Emissions in Forest Soils

Soil pH is the most important controller of denitrification-derived N_2_O emission in the BF (Figure 6C and Table 3), and high N application significantly decreased soil pH (Figure 4). The reasons for soil pH regulating denitrification-derived N_2_O emissions in BF may be as follows. Soil pH can exert a direct and an indirect effect on biological properties (e.g., reductase activities, the abundance, and expression of functional genes, etc.) (Liu et al., 2010; Hu et al., 2015; Han et al., 2019). The reductases for nitrate, nitrite, and nitric oxide are more active at pH <7 (Gillam et al., 2008), while the N_2_O reductase activities are inhibited in soil with low pH (Levy-Booth et al., 2014). A review covering 50 years of research on the effects of soil pH on denitrification also reports that there is a consistent negative relationship between denitrification-derived N_2_O production and soil pH (ŠImek and Cooper, 2002). A close relationship between soil pH and denitrification-derived N_2_O in the BF suggested that soil pH is a strong predictor of denitrifier activity in the BF. In the PF, the abundance of *nirS* is the dominant factor controlling denitrification-derived N_2_O emissions. Previous studies have also reported that the abundance of denitrification genes correlated with *nirS* gene abundance (Morales et al., 2010).

The dominant factors in regulating soil denitrification-derived N_2_O emission were different in these two forests. This may be because differences in the tree species lead to variations in soil environment and the community composition of functional microbes, and changes in these variations induced the differences in the nitrification- and denitrification-derived N_2_O emissions in these acidic forest soils (Barberán et al., 2015; Soper et al., 2018). Moreover, regardless of nitrification- or denitrification-derived N_2_O emissions, soil environmental factors are more important than nitrifier and denitrifier abundance in regulating soil N_2_O emissions in both forests (except denitrification-derived N_2_O emission in PF). Previous studies also demonstrate that tree species-induced changes in community composition and environmental factors play more important roles in regulating soil N_2_O emissions than the abundance of nitrifier/denitrifier functional genes (Qin et al., 2019). In the present study, we still do not know how the species diversity or the nature of individual species affect nitrification- and denitrification-derived N_2_O production in the field, even though several studies have investigated N_2_O emission from plantation forest soils (Arai et al., 2008; Wang et al., 2014; Kou-Giesbrecht and Menge, 2021). Further study needs to focus on the role of the identities of specific species in the production processes of N_2_O under different N inputs.

## Conclusion

Our results demonstrated clearly that nitrification-derived N_2_O emissions decreased with N application rates, while denitrification-derived N_2_O emissions were non-linear responses to N addition. The total soil N_2_O emissions, nitrification-, and denitrification-derived N_2_O emissions were significantly higher in the broadleaf forest than in the conifers forest. Changes in soil environmental factors (i.e., pH and soil NH_4_^+^-N content) induced by N addition are more important than the abundance of nitrification and denitrification functional genes in regulating the production process of soil N_2_O. The incorporation of soil nitrogen substrates, soil pH, and forest type into the nitrogen cycling model will more precisely predict the global N_2_O from soil under elevated N deposition.

## Data Availability Statement

The raw data supporting the conclusions of this article will be made available by the authors, without undue reservation.

## Author Contributions

QQ: conceptualization, methodology, software, investigation, supervision, validation, formal analysis, writing–original draft, writing–reviewing and editing, visualization, data curation, and resources. AM: investigation and writing–reviewing and editing. SJ: writing–original draft, writing–reviewing and editing, visualization, and supervision. YH: conceptualization, methodology, software, validation, formal analysis, writing–original draft, writing–reviewing and editing, visualization, and supervision. All authors contributed to the article and approved the submitted version.

## Conflict of Interest

The authors declare that the research was conducted in the absence of any commercial or financial relationships that could be construed as a potential conflict of interest.

## Publisher’s Note

All claims expressed in this article are solely those of the authors and do not necessarily represent those of their affiliated organizations, or those of the publisher, the editors and the reviewers. Any product that may be evaluated in this article, or claim that may be made by its manufacturer, is not guaranteed or endorsed by the publisher.
