# Supplementary material for: Nitrogen-Induced Changes in Soil Environmental Factors Are More Important Than Nitrification and Denitrification Gene Abundance in Regulating N2O Emissions in Subtropical Forest Soils
Source: Front Plant Sci. 2022 Jul 12;13:950367. doi: 10.3389/fpls.2022.950367 (PMC9315429; doi:10.3389/fpls.2022.950367)
Supplement: Supplementary file 1 [file Table_1.DOCX]

**Supplementary material**

Table S1 Primer pairs and PCR conditions used in real-time qPCR analysis.

| Target genes | Primers | Primer sequences (5’-3’) | | Thermal profile | Reference |
| --- | --- | --- | --- | --- | --- |
| AOA *amoA* | Arch-*amo*AF | STAATGGTCTGGCTTAGACG | | 95°C/2 min; 35 cycles of 95°C/10 s, 55°C/30 s, 72°C/60 s | Francis et al. (2005) |
|  | Arch-*amo*AR | GCGGCCATCCATCTGTATGT | |  |  |
| AOB *amoA* | *amo*A-1F | GGGGTTTCTACTGGTGGT | | 95°C/60 s; 35 cycles of 95°C/10 s, 55°C/30 s, 72°C/60 s | Rotthauwe et al. (1997) |
|  | *amo*A-2R | CCCCTCKGSAAAGCCTTCTTC | |  |  |
| *nirK* | *nir*KF1aCu | ATCATGGTSCTGCCGCG | | 95°C/2 min; 6 cycles of 95°C/30 s, 63-58°C/30 s (-1°C/cycle), 72°C/30 s; 30 cycles of 95°C/30 s, 58°C/35 s; 72°C/45 s | Hallin and Lindgren (1999) |
|  | *nir*KR3Cu | TTGGTGTTRGACTAGCTCCG | |  |  |
| *nirS* | *nir*S1F | CCTA(C/T)TGGCCGCC(A/G)CA(A/G)T | | 95°C/2 min; 35 cycles of 95°C/45 s, 60°C/45 s, 72°C/45 s | Jung et al. (2012) |
|  | *nir*S6R | CGTTGAACTT(A/G)CCGGT | |  |  |
| *nosZ* | *nos*Z2F | CGCRACGGCAASAAG GTSMSSGT | | 95°C/2 min; 6 cycles of 95°C/15 s, 65-60°C/30 s, 72°C/30 s; 30 cycles of 95°C/15 s, 60°C/40 s; 72°C/40 s | Henry et al. (2006) |
|  | *nos*Z2R | CAKRTGCAKSGCRTGGCAGAA | |  |  |
|  |  | |  |  |  |
